# Metabolic reprogramming of clear cell renal cell carcinoma

**DOI:** 10.3389/fendo.2023.1195500

**Published:** 2023-06-06

**Authors:** Haiyan Zhu, Xin Wang, Shihao Lu, Kongbo Ou

**Affiliations:** ^1^ Department of Geriatrics, The First Affiliated Hospital of China Medical University, Shenyang, Liaoning, China; ^2^ Department of Neurology, The First Affiliated Hospital of China Medical University, Shenyang, Liaoning, China; ^3^ Orthopaedics, Changzheng Hospital Affiliated to Second Military Medical University, Shanghai, China; ^4^ Department of Urology, The Third Affiliated Hospital of Soochow University, Changzhou, China

**Keywords:** ccRCC, cancer, metabolism reprogramming, metabolism, glucose, lipids ccRCC, lipids

## Abstract

Clear cell renal cell carcinoma (ccRCC) is a malignancy that exhibits metabolic reprogramming as a result of genetic mutations. This reprogramming accommodates the energy and anabolic needs of the cancer cells, leading to changes in glucose, lipid, and bio-oxidative metabolism, and in some cases, the amino acid metabolism. Recent evidence suggests that ccRCC may be classified as a metabolic disease. The metabolic alterations provide potential targets for novel therapeutic interventions or biomarkers for monitoring tumor growth and prognosis. This literature review summarized recent discoveries of metabolic alterations in ccRCC, including changes in glucose, lipid, and amino acid metabolism. The development of metabolic drugs targeting these metabolic pathways was also discussed, such as HIF-2α inhibitors, fatty acid synthase (FAS) inhibitors, glutaminase (GLS) inhibitors, indoleamine 2,3-dioxygenase (IDO) inhibitors, and arginine depletion. Future trends in drug development are proposed, including the use of combination therapies and personalized medicine approaches. In conclusion, this review provides a comprehensive overview of the metabolic alterations in ccRCC and highlights the potential for developing new treatments for this disease.

## 1 Introduction

Cancer is a disease characterized by uncontrolled cell growth, and to sustain this growth, cancer cells acquire large amounts of energy by altering their metabolic pathways (1, 2). Initially, it was thought that the enhanced glycolysis observed in tumor cells was inefficient in generating energy (3). However, it is now clear that cancer and metabolism are intricately linked, and metabolic reprogramming is a hallmark of cancer. This process involves genetic mutations that alter various metabolic processes, including glucose and lipid metabolism and oxidative phosphorylation (OXPHOS). In other words, cancer cells adapt their metabolism to meet their energy and building block needs (4). The increased glycolysis rate of cancer cells consumes most of the nutrients in the surrounding microenvironment. This metabolic restriction significantly promotes the formation of the tumor microenvironment and reduces the responsiveness of T cells (5).

Renal clear cell carcinoma (ccRCC) is a malignant tumor that arises from the renal tubular epithelium and accounts for 75% of all kidney cancer cases (6, 7). According to the American Cancer Society’s estimates for 2022, approximately 79,000 new cases of kidney cancer will be diagnosed in the United States with ccRCC being the most prevalent subtype (8). This malignant tumor has a poor prognosis, particularly when metastases occur. Surgical resection is often insufficient to manage the disease, and treatment decisions may involve targeted drug therapy, immunotherapy combined with targeted drug therapy, or dual immunotherapy combined (9). Evidence suggests that these therapies can extend the overall and progression-free survival of patients, but they are also associated with the occurrence of adverse events (10, 11).Currently, the International Metastatic Renal Cell Carcinoma Database score (IMDC score) is utilized to guide treatment decisions by providing prognostic stratification.

Metabolic reprogramming is a key feature of ccRCC, and altered gene expressions can lead to significant metabolic changes that fuel tumor growth (12). In particular, ccRCC is associated with abnormalities in glucose metabolism and OXPHOS, as well as unique metabolic alterations such as glutamine addiction (13). The Warburg effect, in which cancer cells preferentially use glycolysis rather than OXPHOS for energy production, is also commonly observed in ccRCC (14).

Understanding these metabolic changes and their impact on tumor growth is essential for developing new and effective treatments for ccRCC. In this article, we provided an overview of ccRCC and the metabolic alterations that occur in the disease, focusing on glucose metabolism, lipid metabolism, and OXPHOS. We also reviewed the pharmacological treatments that have been developed to target these metabolic modifications and discussed potential future directions for drug development in ccRCC.

## 2 Metabolic reprogramming in ccRCC

Metabolic reprogramming provides tumors with a competitive advantage in acquiring survival resources (15). The Warburg effect is a prominent example of metabolic reprogramming in cancer cells, promoting cancer cell proliferation and creating an acidic environment that can facilitate cancer cell migration (16, 17). Glutamine metabolism and enhanced pentose phosphate pathway (PPP) promote macromolecular production and signal transmission in cancer cells (18). Furthermore, the hypoxic signaling pathway can activate neovascularization to facilitate tumor cell proliferation, even in the hypoxic environments surrounding the tumor cells (19). Despite these advantages, modulating the metabolic pathways of cancer cells can limit their metabolic dominance and prevent the development of ccRCC (4).

ccRCC is associated with changes in lipid and glucose metabolism, as evidenced by the presence of the translucent cytoplasm and significant lipid and glycogen accumulation observed in these cells (20).

Most cases of ccRCC arise from mutations in the Von Hippel-Lindau (VHL) allele, which results in altered tumor metabolism in 90% of patients with ccRCC (4). The VHL oncogene, located on the short arm of chromosome 3 encodes the Von Hippel-Lindau(VHL) tumor suppressor protein (pVHL) (21).

Under physiological conditions, pVHL ubiquitinates proline-containing residues in the oxygen-dependent degradation domains (ODDs) of hypoxia-inducible factors (HIFs), promoting their proteasomal degradation (22, 23). HIFs are transcription factors that regulate cellular adaptation to hypoxic environments (24). HIF-1α is the most commonly expressed HIF family member in cells and tissues (25), and its stability is negatively regulated by oxygen levels (26), and cellular metabolism. However, under the pathological hypoxic conditions, HIF-2α is less likely to degrade and instead forms a heterodimer HIF-1β, also known as the aromatic hydrocarbon receptor nuclear transporter (ARNT), that enters the nucleus and activates the transcription of numerous genes (27).

In ccRCC, mutations in VHL result in the accumulation of HIF-2α, which creates a state of “pseudo-hypoxia” (28) and induces metabolic changes, including angiogenesis, epithelial-mesenchymal transition, invasion, and metastatic spread (29). Recent research indicates that HIF-1α functions as a tumor suppressor, while HIF-2α acts as an oncogene in the biology of ccRCC (30).

## 3 Glucose metabolism

Glucose metabolism is a multifaceted process of paramount importance for cellular energy production. The first and foremost step of glucose metabolism is glycolysis, which catalyzes the breakdown of glucose to yield pyruvate (31). Pyruvate is then subjected to aerobic oxidation *via* the tricarboxylic acid (TCA) cycle, leading to the generation of ATP (32), reduced nicotinamide adenine dinucleotide (NADH), and reduced flavin adenine dinucleotide (FADH2) or anaerobic fermentation to produce lactate and ATP (33). In addition, the pentose phosphate pathway (PPP) contributes to the production of glucose for lipid metabolism and nucleic acid synthesis by generating reduced nicotinamide adenine dinucleotide phosphate (NADPH) and ribose 5-phosphate (34).

In ccRCC, glucose metabolism undergoes significant alterations due to the pivotal role played by HIF-1α. HIF-1α stimulates lactate production and reduces pyruvate entry into the mitochondria, leading to diminished TCA cycle activity and ATP production (35). The Warburg effect, a metabolic reprogramming phenomenon observed in cancer cells, is characterized by decreased pyruvate entry into the mitochondria and increased lactate production, regardless of oxygen levels and ATP production efficiency (4). HIF-1α facilitates the expression of BCL2 Interacting Protein 3 (BNIP3), which decelerates mitochondrial metabolic activities (36) and inhibits the enzymatic activity of pyruvate dehydrogenase, thereby hindering the conversion of pyruvate to acetyl coenzyme A and promoting lactate production (37). Additionally, in ccRCC, HIF-1α upregulates the expression of glycolytic enzymes, such as hexokinase (HK), neuron-specific enolase (NSE), phosphoglycerate kinase (PGK), and pyruvate kinase (PK), further corroborating the Warburg effect (38, 39). Among these up-regulated key enzymes, HK2 is one of the key factors involved in the development of a variety of human cancers. Some literature shows that the high expression of HK2 is positively correlated with the advanced tumor, lymph node metastasis and the worst survival rate of renal cancer patients. The high expression of HK2 has been identified as an independent risk factor for RCC; It also shows a positive correlation with immune cell infiltration and prognosis in kidney cancer patients, playing an important role in the occurrence and development of cancer (40).

Glucose metabolism involves the pentose phosphate pathway (PPP), which is a source of NADPH and pentose phosphate (41). The PPP is upregulated in ccRCC to produce more NADPH, which is crucial for maintaining redox homeostasis and protecting against cellular damage caused by reactive oxygen species (ROS) (42). In ccRCC, the PPP is upregulated to produce more NADPH to counteract oxidative stress and lessen the harm caused by excess ROS to the tumor cells (43). Moreover, the pentose phosphate in the PPP also satisfies the high demand for 5-carbon sugars required for nucleotide biosynthesis (44).

In the TCA cycle of ccRCC, enzymes that replenish metabolic fluxes from other pathways are often downregulated (41). Citrate and cis-aconitate levels markedly increase in the TCA cycle of ccRCC, while malate and fumarate levels significantly decrease (13). These latter two decreases are linked to a reduction in succinate dehydrogenase (SDH), leading to a constant depletion of fumarate, and then malate (41), which contradicts the common perception that tumor tissues exhibit high levels of fumarate.

**Figure 1:**
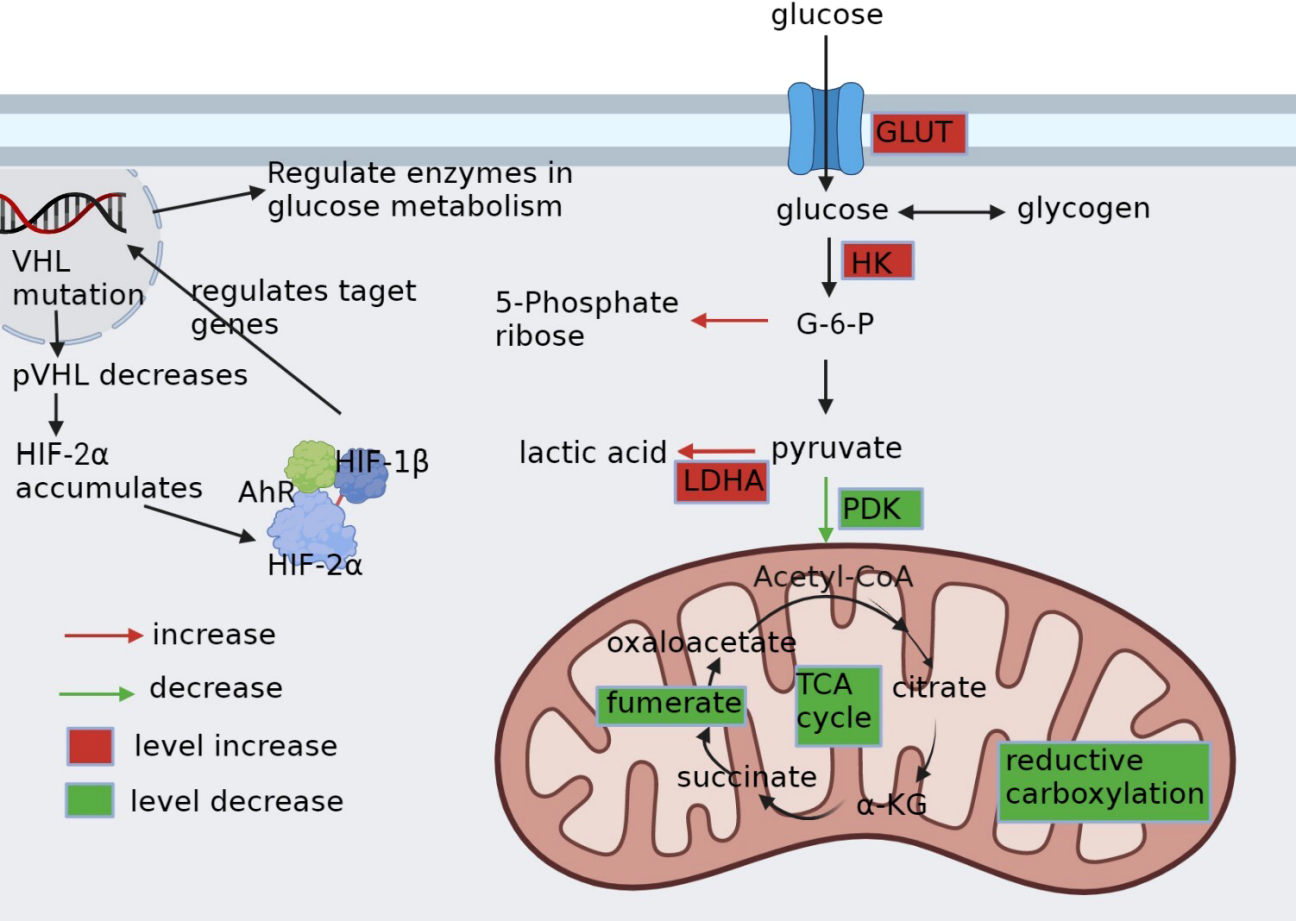
Glucose metabolic reprogramming in ccRCC. pVHL, the product of VHL gene mutation, is reduced. HIF-2α, which depends on pVHL for proteasomal degradation, accumulates intracellularly and binds to HIF-1β and AhR into the nucleus, as well as regulates the expression of carriers and enzyme genes related to glycolysis. It alters the whole glucose metabolism in cancer cells by upregulating glucose transporter protein GLUT, upregulating enzymes HK and LDHA related to glycolysis, downregulating enzyme PDK1 of aerobic glucose metabolism, promoting pentose phosphate pathway, and disrupting metabolites in the tricarboxylic acid cycle.

HIF-1α enhances glucose uptake by activating the glucose transporter protein (GLUT) gene and inhibits mitochondrial respiratory function by controlling the expression of microRNAs such as miR-210 (45). In contrast, HIF-2α in ccRCC primarily controls target genes related to glycolysis while also interact with several important oncogenes (**Figure 1**). Furthermore, it stimulates MYC and P53 activity and boosts the expression of cell cycle regulators (46), thus highlighting its critical role in the development of ccRCC (47). Notably, the selective antagonists of HIF-2α have demonstrated clinical responses in several clinical trials, particularly targeting xenograft tumor models in ccRCC (48).

## 4 Mitochondria metabolism

Recent research results indicate that the Warburg effect promotes tumor growth to a certain extent, but tumor growth is fundamentally dependent on functional mitochondria. In addition to having basic bioenergy functions, mitochondrial metabolism controls redox balance and coordinates cell death. Therefore, mitochondria are promising targets for developing new anticancer drugs (49).

In ccRCC, the TCA cycle undergoes changes. Firstly, there is a reduction in pyruvate entering the mitochondria, followed by changes in key enzymes such as fumarate hydratase (FH) and succinate dehydrogenase (SDH) in the TCA cycle, resulting in imbalanced metabolite content (50). Citrate and aconite levels are significantly higher, while succinate and malic acid saline levels are significantly lower (41). In addition, enzymes that supplement metabolic flux to the TCA cycle through other pathways are typically downregulated (50).

Oxidative phosphorylation (OXPHOS) is a major process in which human cells generate energy in the form of ATP. However, due to changes in the TCA cycle, ccRCC is associated with a decrease in OXPHOS activity (41). The VHL gene mutation in ccRCC leads to the accumulation of HIF-1α, which impairs the oxidation metabolism of glucose and suppresses pyruvate entry into the mitochondria, thereby reducing electron transfer efficiency (13, 51). The downregulation of complex-V and the repression of peroxisome proliferator-activated receptor gamma coactivator-1α (PGC-1α) in ccRCC also contribute to the impaired OXPHOS (52). PGC-1α, a critical transcriptional coactivator of PPARγ, regulates mitochondrial biogenesis and respiration, and its inhibition leads to delayed respiration, reduced mitochondrial transcription factor A (TFAM) expression, and unfavorable prognosis of ccRCC (53).

In addition to HIF-1α, HIF-2α plays a role in the OXPHOS alterations in ccRCC. HIF-2α is associated with the upregulation of antioxidant genes, reducing reactive oxygen species (ROS), decreasing DNA damage from excess ROS, and promoting tumor cell survival (54). These changes in OXPHOS have implications for tumor growth and potential therapeutic targets, including the development of selective HIF-2α antagonists, which have demonstrated clinical efficacy in treating ccRCC (55). Therefore, understanding the mechanisms underlying the impaired OXPHOS in ccRCC could lead to the development of novel treatments for this aggressive cancer.

Mitochondria can promote malignant transformation through three main mechanisms, including (1) mitochondrial reactive oxygen species (ROS) support the accumulation of carcinogenic DNA changes and activation of carcinogenic pathways to some extent;(2) abnormal accumulation in specific mitochondrial metabolites, including fumarate, succinate, and 2-hydroxyglutarate (2HG); (3) functional deficits in MOMP or mitochondrial permeability transition (MPT) are usually necessary for the formation and survival of malignant precursors (49, 56).

Due to the high glycolysis rate of cancer cells and the gradual consumption of oxygen in the local environment, the ratio of NADH to NAD+in cancer cells increases, the redox imbalance, and the production of reactive oxygen species (ROS) increases (57). To prevent the accumulation of ROS, cells can resist oxidation through the thioredoxin system and glutathione system (58). In addition, when ROS levels increase, the main regulatory factor of antioxidant response, the nuclear factor erythroid 2-like 2 (NRF2), stabilizes because its negative regulatory factor, Kelch like ECH related protein 1 (KEAP1), is oxidized and loses its ability to chelate NRF2 in the cytosol for proteasome degradation (57). Excessive ROS production may exceed the antioxidant capacity of cancer cells, therefore cancer cells often upregulate their antioxidant defense mechanisms (59). In ccRCC, HIF-2 α Upregulation of antioxidant genes, reduction of reactive oxygen species (ROS), reduction of excessive ROS damage to DNA, and promotion of tumor cell survival (54).

## 5 Lipid metabolism

**Figure 2:**
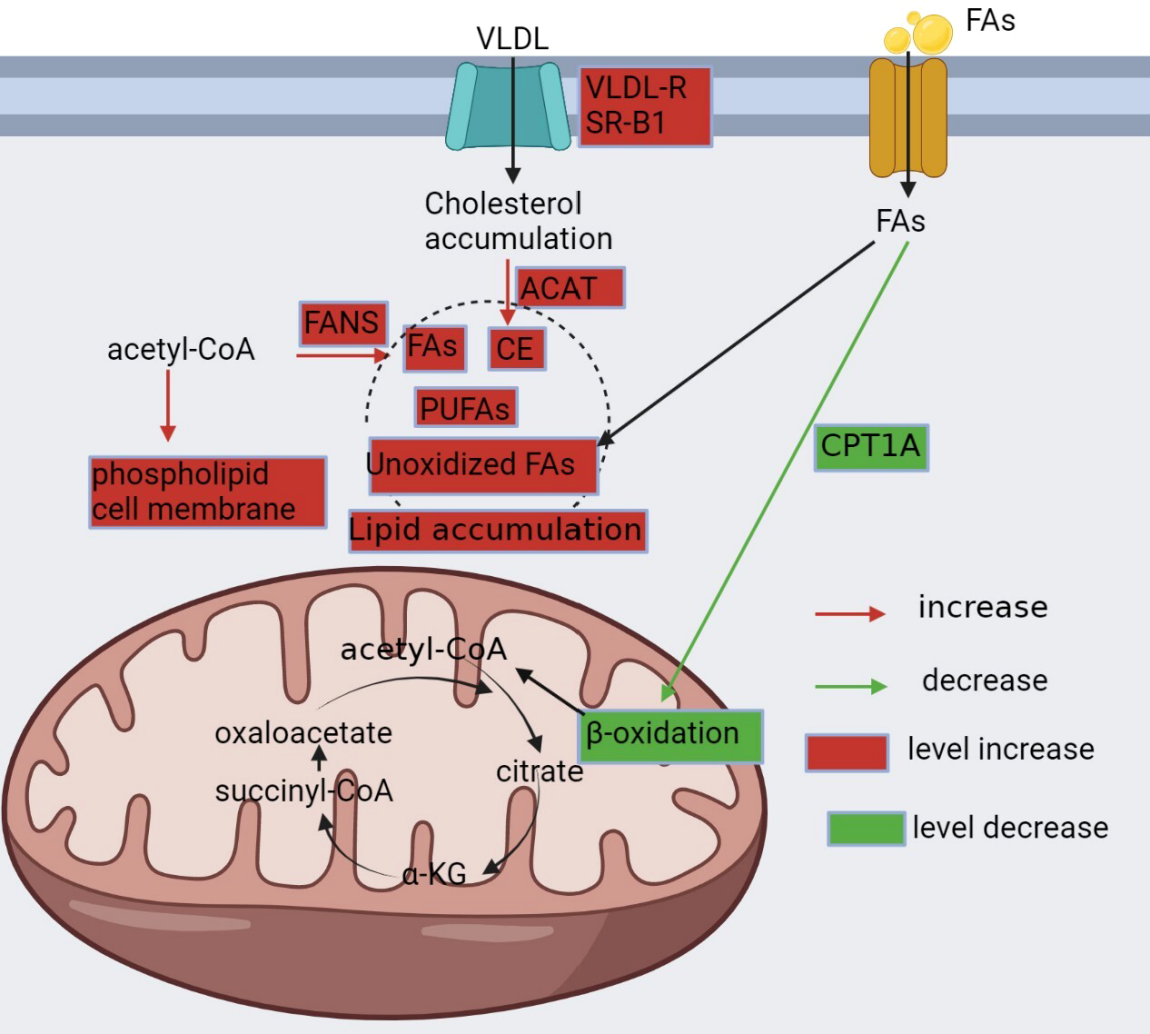
Reprogramming of fatty acid metabolism. In ccRCC, lipid metabolism is altered and characterized by increased synthesis, enhanced utilization, and decreased oxidation to meet the metabolic demands of cancer cells. Renal clear cells upregulate VLDLR and SR-B1 to promote cholesterol uptake, followed by upregulation of ACAT, which converts pure cholesterol into cholesteryl esters; downregulation of CPT1A, a key enzyme in fatty acid β-oxidation, which reduces fatty acid consumption; and upregulation of FASN, which promotes fatty acid synthesis. All of these contribute to the accumulation of lipids in cancer cells.

Lipid metabolism is a functional process pivotal in energy storage and signal transduction (30). Dysregulation of lipid metabolism is a common abnormality in ccRCC (**Figure 2**), which is thought to contribute to the aggressive behavior of the tumor (60). In ccRCC, lipid synthesis and storage are upregulated, while utilization and oxidation are downregulated (61), resulting in the accumulation of cholesterol (62), fatty acids (13), and triglycerides (60). This lipid metabolism reprogramming promotes cell membrane synthesis and cell proliferation while inhibiting the β-oxidation of fatty acids (4). Specifically, increased cholesterol uptake by upregulated lipoprotein receptors, such as the very low-density lipoprotein receptor (VLDL-R) (63) and scavenger receptor B1 (SR-B1) (64), and the expression of acyl-coenzyme A:cholesterol acyltransferase (ACAT) contribute to the accumulation of cholesterol in ccRCC (62). In addition, HIF-2 α activating the expression of hypoxia induced lipid droplet associated protein (HILPDA) and selectively enriching polyunsaturated lipids (65).

Furthermore, ccRCC exhibits fatty acid synthesis due to the upregulation of fatty acid synthase (FAS) (38). However, in contrast to other malignancies, ccRCC displays reduced fatty acid oxidation, which is primarily attributed to the inhibition of carnitine palmitoyltransferase 1A (CPT1A), the key enzyme for fatty acid oxidation (20). In addition, ccRCC shows an increase in the expression of fatty acid desaturase 1 (FADS1), a crucial enzyme in the metabolism of polyunsaturated fatty acids (PUFAs), leading to PUFA accumulation (60).

PUFA is one of the main targets of lipid peroxidation, and lipid peroxidation is a sign of ferroptosis. Studies have identified acyl-CoA synthetase long-chain family member 4 (ACSL4) as a key determinant of iron mortality sensitivity (66). After ACSL4 activation, lysophosphatidylcholine acyltransferase 3 (LPCAT3) is involved in ferrozotic signaling by inserting acyl groups in lysophospholipids, specifically phosphatidylcholine and phosphatidylethanolamine. It is important to note that iron death may also occur in a way that is not dependent on ACSL4. Pharmacologically induced ferroptosis in cancer cells is a promising anticancer strategy, although its role in tumors is still unclear (67).

Lipid metabolism is closely linked to glucose metabolism as glycerol and glucose metabolism are related through glycerol kinase and α-phosphoglycerol dehydrogenase, producing dihydroxyacetone phosphate (34). During glucose overload, acetyl coenzyme A, produced during glucose metabolism, and NADPH and H+ from the PPP are converted into fatty acids by FAS. Phospholipid and ketone body metabolism are also part of lipid metabolism (68). Notably, lipids not only provide energy but also function as signaling molecules that control cell growth and proliferation (30). Thus, targeting lipid metabolism has emerged as a potential therapeutic strategy for ccRCC. Identifying specific enzymes or signaling pathways involved in lipid metabolism could lead to the development of novel therapeutic approaches.

## 6 Amino acid metabolism

### 6.1 Glutamine metabolism

Glutamine is essential for maintaining the redox balance of normal cells by serving as a precursor for the production of α-ketoglutarate (α-KG) and glutathione, which are critical for intracellular redox homeostasis and the synthesis of other amino acids (18). Glutamine is transported into the cells via specialized transporter proteins, such as Solute Carrier Family 1 Member 5 (SLC1A5) (69), and is subsequently converted to glutamate by glutaminase (GLS) (70). In addition to participating in protein synthesis, glutamine also plays various synthetic and metabolic roles in cells, such as promoting the production of nucleotides, hexosamine units, and asparagine, participating in enhancing cellular oxidative stress defense, and consuming many essential amino acids (71). The activity of GLS is positively correlated with the rate of cell growth and malignancy, making it a potential target for anticancer therapies. Inhibition of GLS activity or expression prevent tumor growth (72). The altered cellular metabolism in ccRCC increases glutamine addiction and subsequent cellular pathways (73). The increased demand for glutamine is necessary to support the fast growth and proliferation of malignant cells (73). In addition, high levels of glutamic acid can damage the input of cystine, and can also lead to ROS imbalance and T cell dysfunction, thus forming a tumor microenvironment conducive to tumor survival (5).

In ccRCC, the upregulation of Solute Carrier Family 7 Member 5 (SLC7A5), an amino acid transporter that facilitates glutamine entry into cells, is controlled by HIF-2α, which is a hallmark of this malignancy (74). Once inside the cells, glutamine is converted to glutamate by GLS and further metabolized to α-KG by glutamate dehydrogenase (GDH), serving as the primary mechanism for carbon delivery to the TCA cycle that is essential for cellular lifespan (75). Additionally, the reductive carboxylation of glutamate produces isocitrate, which generates acetyl coenzyme A for lipid synthesis (18, 41).

**Figure 3:**
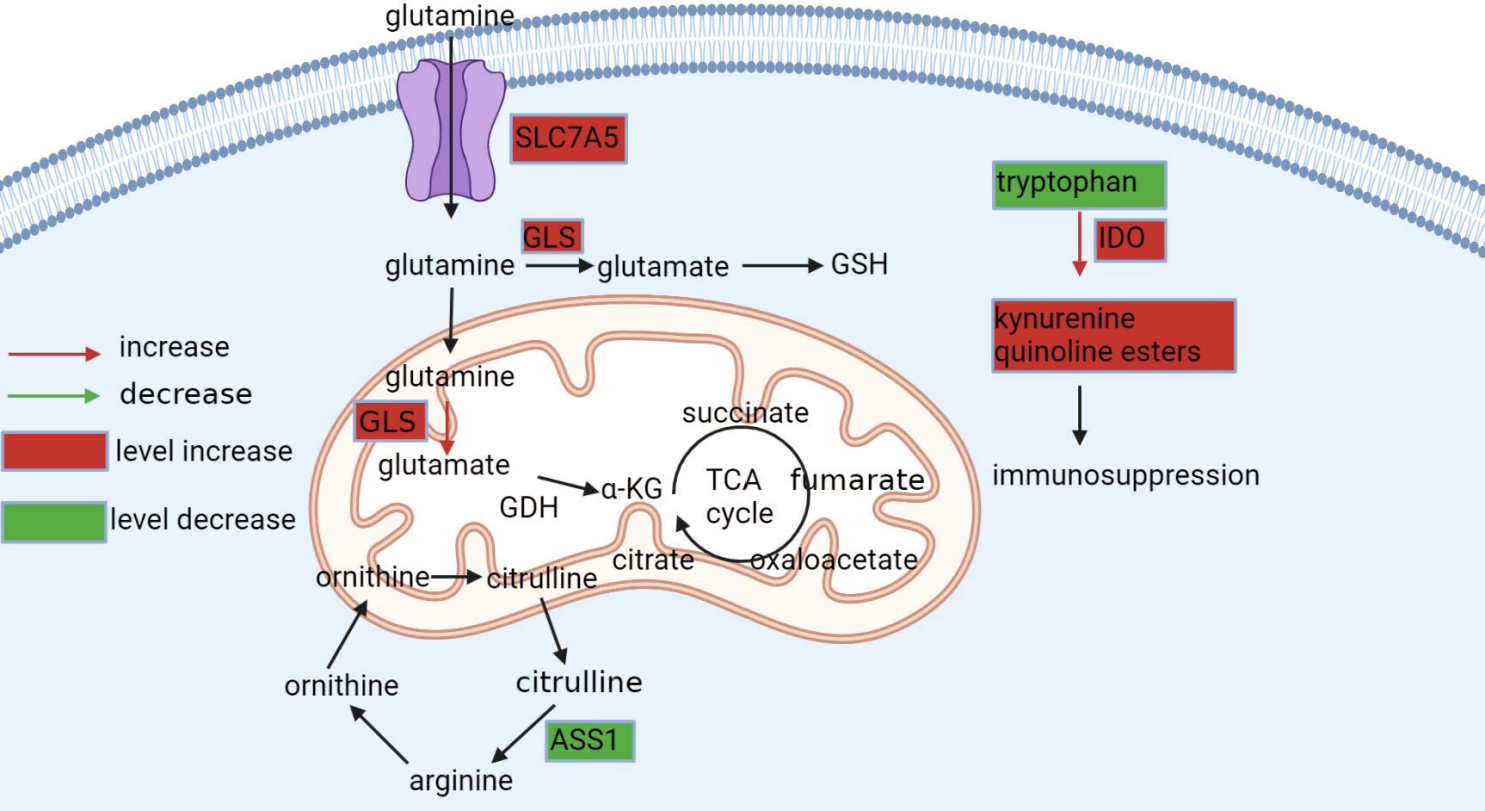
Metabolism of glutamine, arginine, and tryptophan. Glutamine is available intracellularly for various anabolic pathways. Tumor cells exhibit glutamine dependence in ccRCC, upregulating SLC7A5 glutamine transporter protein and GLS enzymes to take up more glutamine and promote its conversion to glutamate, which maintains the redox state *in vivo* or to α-KG, an alternative substrate for the tricarboxylic acid cycle and synthesis of lipids. Tryptophan is degraded in the kynurenine pathway to produce immunosuppressive products, while in ccRCC, this process is upregulated by IOD, leading to the activation of the entire pathway. Arginine is synthesized from citrulline *via* ASS1, but AAS1 is not expressed or downregulated in ccRCC and cannot synthesize this amino acid and can only be taken up from the blood.

Elevated glutamine levels in ccRCC are associated with enhanced glutamate production, which is a crucial mechanism for neutralizing ROS (76). Glutamine also promotes glycolysis, cell proliferation, and immortalization while decreasing cancer cell death by inhibiting the expression of thioredoxin-interacting protein[]. Therefore, the altered glutamine metabolism in ccRCC highlights its potential as a therapeutic target for treating this malignancy. **Figure 3** illustrates the glutamine metabolism pathway.

### 6.2 Tryptophan metabolism

Tryptophan plays a vital role in promoting T cell-mediated immune response against tumors. However, the excessive oxidation of tryptophan to kynurenine pathway metabolites leads to T-cell disfunction, allowing tumors to evade immune surveillance. Moreover, kynurenine pathway inhibit T-cell activation (77). In tumor-draining lymph nodes, overactive indoleamine 2,3-dioxygenase (IDO) promotes dendritic cells to directly suppress and inhibit T cells, thus impairing antigen responses and recognition (78).

In ccRCC, the dysregulation of immune checkpoint molecules has been linked to elevated indoleamine 2,3-dioxygenase (IDO) expression, which results in decreased levels of tryptophan and activation of the kynurenine pathway. This metabolic reprogramming promotes tumor cell proliferation by hindering the effectiveness of IFN-α therapy and inducing immunosuppression (79, 80). Furthermore, IDO overexpression is strongly associated with cancer metastasis. *In vitro* experiments utilizing lung cancer cells demonstrate that IDO overexpression increases cell viability, while IDO knockdown decreases cell viability. In animal models, injecting human lung cancer cells that overexpress IDO into mice increases metastases in the brain, liver, and bone (81) (**Figure 3**). These findings indicate that IDO may serve as a potential therapeutic target for ccRCC and other malignancies, highlighting the need for further research in this area.

### 6.3 Arginine metabolism

In ccRCC, alterations in arginine metabolism can be observed, including changes in arginine transporters and enzymes such as arginase and arginine succinate synthase 1 (ASS1). Tumor cells often display a downregulation or absence of ASS1, the enzyme responsible for synthesizing arginine from citrulline. This leads to a dependence on exogenous arginine for cancer cell survival, which has been confirmed by proteomic profiling of biopsy samples from patients with ccRCC (**Figure 3**). Therefore, targeting arginine metabolism may be a viable strategy to inhibit cancer growth by depriving cancer cells of arginine[]. Moreover, studies have identified arginine deprivation as a promising therapeutic approach to induce selective cytotoxicity in ASS1-deficient tumors (82). Therefore, a better understanding of the altered arginine metabolism in ccRCC can provide insights into potential therapeutic targets for treating this cancer (83).

## 7 Treatment

ccRCC is frequently associated with genetic mutations that lead to hypoxic alterations, with the most commonly mutated gene being VHL (84). VHL mutations lead to the intracellular accumulation of HIF-α, which in turn upregulates the expression of vascular endothelial growth factors (VEGFs) (19).

Previously, the main therapeutic approach for ccRCC was to target angiogenesis using VEGF receptor (VEGFR) or VEGF inhibitors like sunitinib (85). However, these inhibitors have limited efficacy and can cause adverse effects such as vascular toxicity and off-target effects (86).

**Figure 4:**
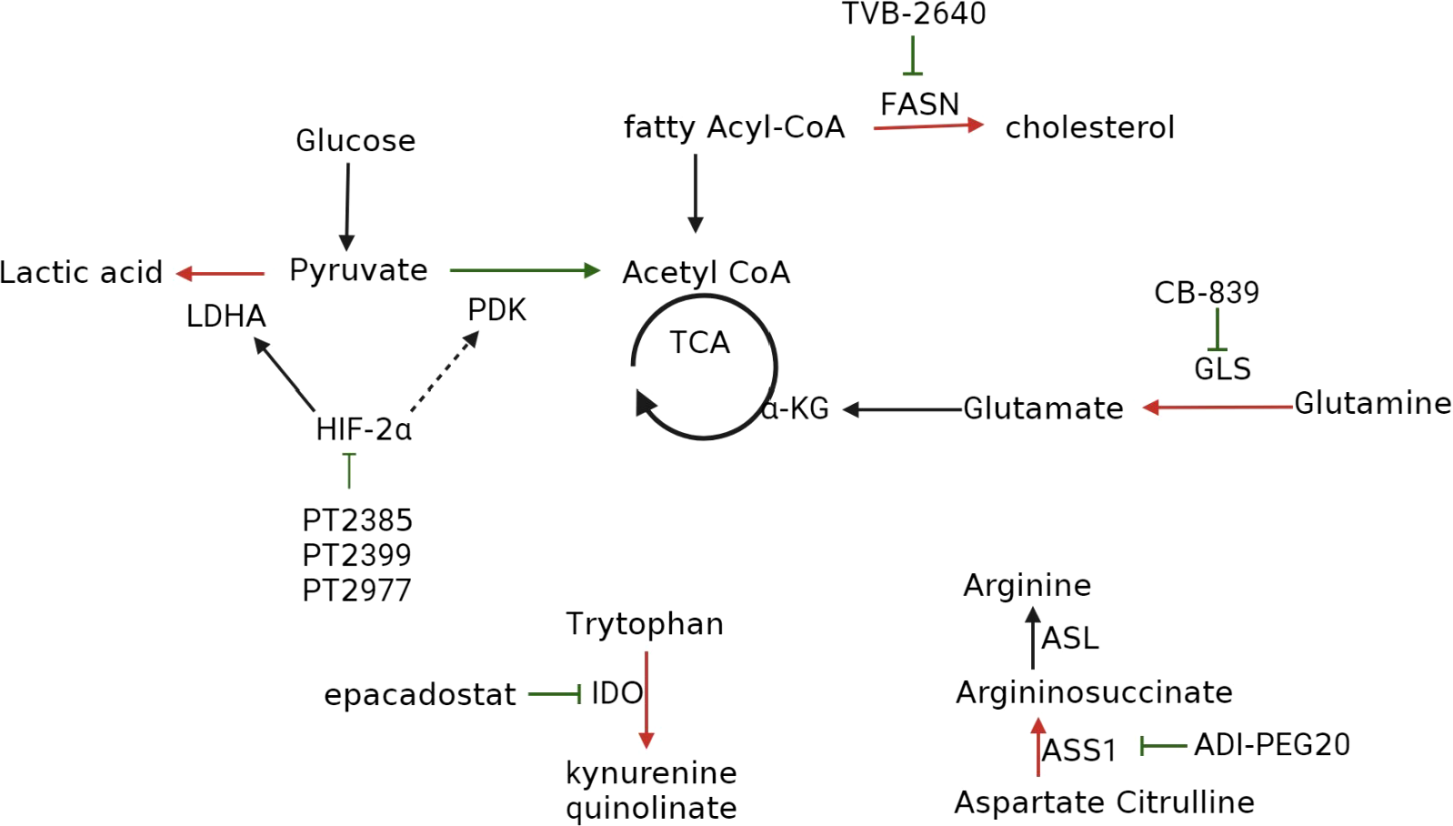
Inhibitors that target metabolic reprogramming in ccRCC. Glycolysis can be inhibited with HIF-2α inhibitors such as PT2385, PT2399, and PT2977; FAS can be suppressed with FAS inhibitor TVB-2640; tryptophan metabolism can be inhibited with IDO inhibitors epacadostat, navoximod, KHK2455, LY3381916, MK-7162; glutamine metabolism can be inhibited using the GLS inhibitor CB-839; extracellular arginine can be depleted using ADI-PEG20.

As ccRCC is characterized by metabolic reprogramming, targeting specific enzymes or proteins implicated in dysregulated metabolic pathways has shown promise in developing drugs that can selectively kill tumor cells with minimal adverse effects on normal cells (87). Among these metabolic changes, the most classic is the increase in glycolysis. In ccRCC, we can use glycolysis inhibitors to inhibit tumor cells, following the treatment methods of hepatocellular carcinoma (15). Unfortunately, although early clinical studies have shown that targeting the glycolysis pathway as a treatment method to inhibit cancer progression is effective, there have been no clinical trials to confirm it (88), so there is no detailed explanation in this article. **Table 1** and **Figure 4** provide an overview of the specific enzymes and proteins targeted in this manner. Focusing on these dysregulated metabolic pathways offers the potential to develop more effective and better-tolerated treatment options for patients with ccRCC (41, 87).

**Table 1:** Metabolic drug for ccRCC.

Classification	name of drug	expericence classification
HIF-2 α inhibitor	PT2399	Not yet in progress
	PT2385	clinical trail phasel
	PT2977	clinical trail phasel, 2
FAS inhibitors	C75	Not yet in progress
	TVB-2640	clinical trail phase (not ccRCC)
Glutaminase inhibitors	CB-839	Not yet in progress
IDO inhibitors	epacadostat	clinical trail phasel
	navoximod	clinical trail phasel
	KHK2455, LY3381916, MK-7162	Not yet in progress
Arginine loss	ADI-PEG20	clinical trail phase3

### 7.1 HIF-2α inhibitor

Targeting the HIF-2α pathway represents a promising approach for treating ccRCC, as HIF-2α is a key downstream effector of the VHL tumor suppressor protein, which is frequently mutated in ccRCC (47, 89). HIF-2α promotes tumorigenesis and metastasis by regulating angiogenesis, cell proliferation, and metabolism, making it an attractive therapeutic target.

Although the HIF-2α pathway was once considered “undruggable” (90), recent technological advancements have revealed a structural defect in HIF-2α, specifically PAS-B, leading to the development of first-generation HIF-2α antagonists, including PT2399 and PT2385. These antagonists induce a conformational change in PAS-B, thereby impeding the formation of the HIF-2α/HIF-1β heterodimer, and represent a promising avenue for the treatment of ccRCC (91).

PT2399 has exhibited superior activity to sunitinib and has been effective against sunitinib-resistant tumors. However, its long-term use can lead to drug resistance through mutations in the HIF-2α binding site or HIF-1β second site inhibition mutations (92). Moreover, PT2399 does not affect the expression of HIF-2α target genes, which can prevent the segregation of HIF-2α and maintain the expression of the HIF-2β gene. In contrast, PT2385 has demonstrated encouraging results in a phase I trial, exhibiting good tolerance with no dose-limited toxicity or discontinuation of treatment due to adverse events. Remarkably, 2%, 12%, and 52% of patients achieved complete, partial, and stable remissions, respectively (48, 93).

Second-generation HIF-2α inhibitors, such as PT2977 (MK-6482, belzutifan), have been developed to overcome some of the limitations of first-generation compounds (94). PT2977 binds to HIF-2α at a site adjacent to the PAS-B domain and induces a conformational change that prevents HIF-2α from binding to its target genes. PT2977 has demonstrated excellent pharmacological properties, including low lipophilicity, high oral bioavailability, and a favorable safety profile. In a phase II clinical trial, patients with ccRCC who received 120 mg of PT2977 orally per day had an objective response rate of 49%. The main adverse events reported were grade 1 and grade 2, which can be managed with appropriate measures (95, 96).

In summary, selective HIF-2α antagonists a promising therapeutic strategy for treating ccRCC. Second-generation inhibitors such as PT2977 have shown improved efficacy and safety compared with first-generation compounds, highlighting their potential as a new class of anticancer agents.

### 7.2 FAS inhibitors

ccRCC is characterized by specific genetic mutations that dysregulate cellular processes, including lipid metabolism. In particular, upregulation of FAS expression in ccRCC increases fatty acid levels that fuel the cancer cells and post-translationally modify proteins. Fatty acids also play a crucial role in maintaining redox homeostasis and energy levels, which are vital for tumor cell growth and survival (97). Given the importance of lipid metabolism in ccRCC, limiting the production of fatty acids is an effective strategy for treating this type of cancer.

Studies have shown a positive correlation between FAS expression and tumor aggressiveness and a negative correlation with prognosis in ccRCC (98). Hence, the use of FAS inhibitors could be a promising treatment strategy for ccRCC. Preclinical experiments have demonstrated the inhibitory effect of C75, a FAS inhibitor, on the aggressiveness and proliferation of ccRCC (99).

TVB-2640 is a noval FAS inhibitor that has shown great promise in clinical studies (100). In a phase I clinical trial, TVB-2640 effectively reduced fatty acid production in patients with non-small cell lung cancer. In subsequent clinical trials for breast and ovarian cancer, TVB-2640 exhibited favorable clinical activity and safety, with no gastrointestinal or serum toxicity observed (101, 102). Adverse events were mainly mild and included skin and eye effects, which were manageable with appropriate measures. TVB-2640 is currently being evaluated in multiple ongoing clinical trials for various types of cancer, including ccRCC.

Overall, these findings suggest that FAS inhibitors such as TVB-2640 represent a promising therapeutic approach for ccRCC, and ongoing clinical trials will provide further insight into their effectiveness and safety.

### 7.3 Glutaminase inhibitors

Glutamine plays a critical role in energy production, maintenance of redox stability, and macromolecule synthesis in cancer cells, making it an attractive target for clinical cancer treatment. In ccRCC, GLS functions as a compensatory mechanism to restore the TCA cycle and stimulate cell proliferation to a lesser extent (4). CB-839 is a GLS inhibitor that has shown promising results in preclinical studies. When combined with Everolimus, an mTOR inhibitor commonly used in the treatment of ccRCC, it has been shown to enhance antitumor activity in animal models. While there is a lack of clinical trials exploring this potential treatment strategy for ccRCC, the preclinical data suggests that it could be an effective approach (103).

Therefore, it is important to conduct clinical trials to explore the effectiveness and safety of combining CB-839 with Everolimus for the treatment of ccRCC. Such trials will provide valuable insights into the potential of this treatment strategy and whether it can be used as an effective therapeutic approach for ccRCC patients.

### 7.4 IDO inhibitors

IDO is an enzyme that plays a role in the catabolism of tryptophan *via* the kynurenine pathway. By depleting tryptophan and activating T cells, IDO inhibits the immunosuppressive effect in the local tumor microenvironment and suppresses antitumor T cells, thereby promoting tumor metastasis (80). Therefore, IDO has emerged as a potential therapeutic target for cancer treatment.

Epacadostat, a selective IDO-targeted inhibitor, has shown promising results in preclinical trials by improving the lysis of tumor antigen-specific T cells (104). However, its efficacy in clinical trials has been disappointing, with some studies reporting adverse effects such as toxicity and lack of efficacy. Although phase I clinical trials have demonstrated promising antitumor activity against various advanced solid tumors with the combination of Epacadostat and the PD-1 inhibitor pembrolizumab, further investigation is necessary to determine its optimal use and potential limitations (93, 105).

Navoximod is another IDO inhibitor. It is well tolerated at a dose of 800 mg BID, with rapid absorption and moderate bioavailability. Despite its promising pharmacokinetic profile, Navoximod as a monotherapy has demonstrated only modest efficacy against tumors (106, 107). However, a combination therapy of Navoximod and Atezolizumab has demonstrated acceptable safety and observed antitumor activity. Nonetheless, it remains uncertain whether adding Navoximod to Atezolizumab confers any additional benefits to patients (108).

To fully activate the host immune system, a combined regimen that involves one or more specific immunotherapeutic agents may be required, as IDO inhibitors have demonstrated limited efficacy as monotherapies (106). Several other IDO inhibitors, such as KHK2455, LY3381916, and MK-7162, are undergoing clinical trials to assess their safety, tolerability, and antitumor activity (78). These agents have different mechanisms of action and pharmacokinetic profiles, which may result in improved clinical outcomes when combined with other immunotherapeutic drugs. Further investigations are needed to fully elucidate the optimal combinations and dosing regimens for these agents in different cancer types and patient populations.

### 7.5 Arginine loss

Arginine dystrophy is a metabolic alteration in some tumors where cells become addicted to extracellular arginine supply due to the absence or low levels of ASS1 (109, 110). Arginine is essential for metabolic pathways, including nitric oxide synthesis and protein biosynthesis (111). In ccRCC, the use of polyethylene glycol form of arginine deaminase (ADI-PEG20) can lower circulating arginine levels by catabolizing arginine to citrulline, thereby limiting tumor growth. However, this therapeutic approach may be limited by the re-expression of ASS1 (4).

Clinical trials have demonstrated the safety, tolerability, and clinical efficacy of ADI-PEG20 in reversing medication resistance in patients with arginine-dystrophic tumors (112). Furthermore, promising results have been reported in clinical studies evaluating ADI-PEG20 as a potential therapy for various cancer types, such as non-small cell lung cancer, acute myeloid leukemia, and uveal melanoma (113, 114).

Considering the potential of arginine depletion as a therapeutic strategy, further studies are warranted to optimize its clinical use in combination with other cancer therapies and investigate the mechanisms of resistance to this approach.

## 8 Conclusions

Clear cell renal cell carcinoma (ccRCC) is characterized by significant metabolic reprogramming, leading to altered energy requirements and redox homeostasis. Cancer cells predominantly utilize anaerobic glycolysis and HIF-driven lactate metabolism, while efficient TCA cycling is downregulated. NADPH synthesis in the PPP is upregulated to protect against ROS and nucleotide damage. The high lactate content of the tumor microenvironment promotes immunosuppression and migration, while tryptophan breakdown leads to increased synthesis of immunosuppressive kynurenine metabolites. Lipid synthesis and utilization are elevated, whereas lipid oxidation is suppressed in ccRCC. However, glutamine absorption is increased to produce fatty acids and counteract oxidative stress and ROS. Further research is needed to fully understand the complex metabolic alterations in ccRCC and develop effective therapeutic strategies targeting these pathways.

The development of drugs that specifically target abnormal metabolism represents a promising avenue for the treatment of ccRCC, which currently has limited therapeutic options in clinical practice. Targeting enhanced or altered metabolic pathways that selectively affect proliferating cancer cells while sparing normal cells is a feasible strategy for the development of novel therapies for ccRCC, given its unique metabolic properties.

However, there are potential drawbacks to targeting metabolic pathways, including the possibility of affecting other rapidly proliferating cells, and the effectiveness of antimetabolic drugs is related to cancer cell mutation patterns.

Nonetheless, the benefits of developing antitumor cell metabolism drugs outweigh the drawbacks. Anti-metabolites may offer a more favorable safety profile for ccRCC compared with traditional anti-angiogenic medications, potentially reducing cardiovascular side effects. Furthermore, metabolomics can aid in the discovery of targeted treatments for ccRCC. With the anticipated emergence of new techniques in novel metabolism, we expect to see an increase in therapeutic options for treating ccRCC in the near future.

## Author contributions

HZ wrote the manuscript. XW and SL made a revised version. All authors contributed to the article and approved the submitted version.

## Glossary

**Table:**

ccRCC	clear cell renal cell carcinoma
IMDC	International Metastatic Renal Cell Carcinoma Database
PPP	pentose phosphate pathway
VHL	Von Hippel-Lindau
pVHL	Von Hippel-Lindau tumor suppressor protein
HIF	hypoxia-inducible factor
NADH	reduced nicotinamide adenine dinucleotide
FADH	reduced flavin adenine dinucleotide
PDK1	pyruvate dehydrogenase kinase 1
BNIP3	BCL2 Interacting Protein 3
GLUT	glucose transporter protein
TFAM	mitochondrial transcription factor A
ACAT	Acyl coenzyme A-cholesterol acyltransferase
CE	cholesteryl ester
SR-B1	scavenger receptor B1
VLDL	very low-density lipoprotein receptor
FAS	Fatty acid synthase
CPT1A	carnitine palmitoyltransferase 1A
FADS1	Fatty acid desaturase 1
PUFA	polyunsaturated fatty acid
GLS	Glutaminase
GDH	glutamate dehydrogenase
IDO	indoleamine 2,3-dioxygenase
TDO	tryptophan-2,3-dioxygenase
ASS1	arginine succinate synthase 1
VEGF	vascular endothelial growth factor
VEGFR	vascular endothelial growth factor receptor
OXPHOS	oxidative phosphorylation
HK	hexokinase
NSE	neuron-specific enolase
PGK	phosphoglycerate kinase
PK	pyruvate kinase
ROS	reactive oxygen species
NRF2	nuclear factor erythroid2-related factor 2
KEAP1	kelch-like ECH-associated protein-1
SDH	succinate dehydrogenase
PGC-1α	PPARγ coactivator-1α
FA	fatty acid
SLC1A5	Solute Carrier Family 1 Member 5
SLC7A5	Solute Carrier Family 7 Member 5
α-KG	α-ketoglutarate
